# Radiological distribution patterns in restrictive chronic lung allograft dysfunction: Impact on survival across all phenotypes

**DOI:** 10.1016/j.jhlto.2025.100232

**Published:** 2025-02-18

**Authors:** Taiki Fukuda, Yusei Nakamura, Shu-Chi Tseng, Yuki Ko, Staci M. Gagne, Takeshi Johkoh, Yi Li, David C. Christiani, Hiroya Ojiri, Lynette Sholl, Mizuki Nishino, Hiroto Hatabu

**Affiliations:** a Center for Pulmonary Functional Imaging, Department of Radiology, Brigham and Women’s Hospital and Harvard Medical School, Boston, Massachusetts; b Department of Radiology, The Jikei University School of Medicine, Tokyo, Japan; c Department of Clinical Radiology, Graduate School of Medical Sciences, Kyushu University, Fukuoka, Japan; d Department of Medical Imaging and Intervention, Chang Gung Memorial Hospital at Linkou and Chang Gung University, Taoyuan City, Taiwan; e Department of Imaging, Dana-Farber Cancer Institute, Boston, Massachusetts; f Department of Radiology, Kansai Rosai Hospital, Hyogo, Japan; g Department of Biostatistics, University of Michigan, Ann Arbor, Michigan; h Division of Pulmonary and Critical Care Medicine, Department of Medicine, Massachusetts General Hospital and Harvard Medical School, Boston, Massachusetts; i Department of Environmental Health, Harvard TH Chan School of Public Health, Boston, Massachusetts; j Department of Pathology, Brigham and Women’s Hospital and Harvard Medical School, Boston, Massachusetts

**Keywords:** lung transplant, chronic lung allograft dysfunction, restrictive allograft syndrome, prognosis, computed tomography

## Abstract

**Background:**

Restrictive chronic lung allograft dysfunction (CLAD) demonstrates poor outcomes after lung transplantation. However, the impact of radiological patterns on survival within a restrictive CLAD under the new International Society for Heart and Lung Transplantation (ISHLT) criteria remains unclear.

**Methods:**

We analyzed retrospectively 241 bilateral lung transplant recipients between 2005 and 2021. CLAD was diagnosed and classified per the 2019 ISHLT criteria. Restrictive phenotype included restrictive allograft syndrome (RAS) and mixed phenotype. In these cases, RAS-like opacities (RLOs) were evaluated both qualitatively and semiquantitatively on computed tomography at CLAD diagnosis. RLOs were classified into upper-predominant and diffuse/lower-predominant distribution groups. Overall survival after CLAD diagnosis was assessed using Kaplan-Meier method with log-rank test and Cox proportional hazards models.

**Results:**

Eighty-three patients were diagnosed with CLAD after transplantation. Twenty-one (25.3%) had restrictive phenotype, which showed shorter survival compared to bronchiolitis obliterans syndrome (median survival: 19.8 vs 68.1 months; hazard ratio [HR], 4.53; 95% confidence interval [CI], 1.96-10.49; *p* < 0.001). Within the restrictive phenotype, the upper-predominant group demonstrated longer survival than the diffuse/lower-predominant group (median survival: 61.1 vs 15.5 months; *p* = 0.008). The diffuse/lower-predominant group had shorter survival compared to any other CLAD phenotype (HR, 8.45; 95% CI, 3.40-21.04; *p* < 0.001). The extent of RLOs within each distribution pattern was not significantly associated with survival.

**Conclusions:**

In restrictive phenotype CLAD, RLO distribution patterns determined survival outcomes, with diffuse/lower-predominant showing the poorest prognosis, while the extent of RLOs within each pattern did not correlate with prognosis.

## Background

Lung transplantation is the ultimate treatment option for end-stage lung diseases. Advances in immunosuppression therapy and surgical techniques have improved patients' survival after transplantation, while long-term survival in lung transplantation remains limited compared to other solid-organ transplantations.^1^ Chronic lung allograft dysfunction (CLAD) remains a major obstacle,^2^ affecting 50% of lung transplant recipients within 5 years of operation.^3^, ^4^ CLAD manifests primarily in 2 forms: bronchiolitis obliterans syndrome (BOS) and restrictive allograft syndrome (RAS). Addressing the previous lack of standardization, the International Society for Heart and Lung Transplantation (ISHLT) established a consensus document for CLAD diagnosis and phenotype classification in 2019.^5^

In addition to spirometry-based CLAD diagnosis, the new ISHLT criteria now include computed tomography (CT) image evaluation for RAS diagnosis.^5^ Specifically, persistent opacities required for the diagnosis of RAS, originally termed RAS-like opacities (RLOs), include ground-glass opacities (GGOs), consolidation, and small linear and reticular opacities.^5^, ^6^ These abnormalities may manifest as multilobar involvement and/or progressive pleural thickening, indicating pulmonary and/or pleural fibrosis consistent with a restrictive physiological pattern.^5^ Consequently, CT has emerged as a valuable complementary tool for precise phenotype characterization of CLAD,^7^ providing objective evaluation regardless of patients’ effort or physical conditions.

RAS demonstrates a significantly worse prognosis compared to BOS.^8^ Notably, Sato et al reported widely varying survival rates among RAS patients (490 ± 417 days; *n* = 25),^9^ which may indicate a heterogeneous nature among the RAS population. Under the new ISHLT criteria, mixed phenotype, which exhibits obstructive ventilatory defects, has now been recognized alongside RAS, as both share the diagnostic criteria of restrictive ventilatory defects with RLOs.^5^ Importantly, both RAS and mixed phenotypes have been reported to result in poorer prognosis compared to BOS.^6^ Although RAS has been recognized as a severe phenotype of CLAD, existing literature has focused predominantly on spirometry-based definitions, often underestimating the prognostic value of radiological findings.

Previous studies^10^, ^11^ have demonstrated that the distribution of RLOs on CT correlates with survival outcomes, and Dubbeldam et al^11^ have provided valuable insights into varying progression patterns of opacities in restrictive phenotype cases with different prognoses. While these distribution patterns offer important prognostic information, the potential impact of the degree of radiological involvement remains to be fully explored.

Building upon these findings, we aimed to further investigate the overall survival (OS) differences among phenotypes of CLAD with new ISHLT criteria based on CT images at diagnosis, focusing on both the distribution patterns of RLOs and evaluating whether the extent of these opacities impacts survival outcomes in restrictive phenotype.

## Materials and methods

### Study design

This study was a single-center retrospective cohort analysis approved by the Mass General Brigham institutional review board (No. 2021P002789), a waiver of informed consent, and in compliance with the ISHLT ethics statement. All lung transplant recipients at our institution between January 2005 and December 2021, based on the pathology database, were included in the initial review.

To evaluate CLAD, the following inclusion criteria were used: (1) patients undergoing first bilateral lung transplantation or heart-lung transplantation; (2) post-transplant survival exceeding 3 months; and (3) availability of sufficient follow-up data for CLAD diagnosis, defined as: pulmonary function tests (PFTs) data including baseline post-transplant measurements and serial follow-up measurements for CLAD diagnosis, persistent opacities documented on chest imaging (CT and/or chest x-ray) at the time of CLAD diagnosis, chest CT obtained within 3 months of CLAD diagnosis for phenotype classification. Clinical, radiological, and pathological data were obtained from medical records. All eligible patients were followed until September 2024.

### CLAD definition

According to the ISHLT criteria,^5^ CLAD was diagnosed as a sustained and irreversible decline in forced expiratory volume in 1 second (FEV_1_) to ≤80% of the post-transplant baseline, confirmed by 2 measurements taken at least 3 weeks apart. The baseline was defined as the average of the 2 highest post-transplant FEV_1_ values. Only cases that survived over 3 months following CLAD diagnosis were included. This definition was applied after excluding other etiologies, such as heart failure and transient infection.

### CLAD phenotype classification

Based on the new ISHLT criteria, CLAD was categorized into 5 phenotypes: BOS, RAS, mixed, undefined, and unclassified.^5^ BOS was defined as PFTs consistent with an obstructive ventilatory defect (FEV_1_/forced vital capacity (FVC) <0.7) without persistent opacities, namely RLOs; multilobar opacities (GGOs, consolidation, or reticular opacities) and/or progressive pleural thickening, suggesting fibrosis on chest imaging.^5^ RAS was defined as a total lung capacity (TLC) ≤90% of baseline (baseline as the average of the 2 measurements obtained at the time or near the best 2 post-transplant FEV_1_ measurements but before CLAD onset) with RLOs on chest imaging. We used FVC ≤80% compared to baseline as a surrogate marker of TLC.^5^, ^12^ Mixed phenotype was defined as CLAD with PFTs that exhibits a combination of obstructive and restrictive ventilatory defects (FEV_1_/FVC <0.7 and FVC ≤80% of baseline, respectively) with RLOs. Undefined phenotype included cases with obstructive defects and RLOs but no FVC decline or combined obstructive and restrictive decline in PFTs without RLOs. Unclassified phenotype comprised cases not meeting criteria for other phenotypes.

In the present study, while CLAD encompasses 5 phenotypes according to the new ISHLT criteria (BOS, RAS, mixed, undefined, and unclassified), we combined RAS and mixed phenotypes as a “restrictive phenotype” group since both demonstrate restrictive ventilatory defects with RLOs based on the diagnostic criteria. All CLAD phenotypes were classified at the time of CLAD diagnosis and reassessed 3 months post diagnosis.^5^

### Radiological and pathological assessment of restrictive phenotype

Two thoracic radiologists (with 5 and 12 years of chest imaging experience) independently reviewed chest CT images of patients who met the RAS diagnostic criteria based on PFTs. CT images were reconstructed with 1-mm axial thickness and displayed using lung window settings on inspiration (level, −600 Hounsfield unit and width, 1,500 Hounsfield unit). RLOs, including GGOs, consolidation, and reticular opacities, were evaluated according to the Fleischner Society nomenclature.^13^ The radiologists assessed (1) the distribution pattern of RLOs (upper-predominant, lower-predominant, and diffuse distribution, with the pulmonary hilum serving as the boundary) and (2) performed semiquantitative analysis of these RLOs using a 6-point scale for upper and lower lung zones separately (0, no involvement; 1, less than 5% involvement; 2, 5%-25% involvement; 3, 26%-49% involvement; 4, 50%-75% involvement; and 5, greater than 75% involvement). Upper and lower RLO scores were calculated by summing the areas affected by any type of RLO in the respective lung zones. The total RLO score was obtained by adding the scores from both zones (range 0-10). Any discrepancies in the evaluation were resolved through consensus-based discussions with a third reviewer (with more than 20 years of experience). Sequential follow-up CT images were reviewed from CLAD diagnosis until retransplantation, death, or end of the study based on RLOs distribution patterns. For the analysis of radiological progression patterns, only patients with at least 1 follow-up CT scan after CLAD diagnosis were included.

Available pathological specimens from autopsy and transbronchial biopsy (TBB) were initially examined by thoracic pathologists and confirmed by a senior thoracic pathologist with more than 20 years of expertise in thoracic pathology to evaluate CLAD-associated histopathological changes, focusing particularly on findings reported in restrictive phenotype (including pleuroparenchymal fibroelastosis [PPFE], acute fibrinous organizing pneumonia [AFOP], and diffuse alveolar damage [DAD]).^14^, ^15^, ^16^, ^17^ These findings were correlated with radiological results.

### Statistical analysis

Demographic characteristics were assessed using counts, percentages, means ± standard deviations, and median with interquartile range (IQR). Mann-Whitney U test, Kruskal-Wallis test, chi-square test, and Fisher exact test were used for group comparisons as appropriate. Survival analyses of the time between CLAD diagnosis and death (OS) were conducted using Kaplan-Meier curves and a log-rank test. Multivariable Cox proportional hazard models were performed, with age and sex included a priori based on their established associations with post-transplant survival.^18^, ^19^ Univariate Cox proportional hazard models were used within each distribution pattern group to evaluate the association between total RLO scores and survival. Survival was measured in months from CLAD diagnosis until death, retransplantation, or end of study. Median follow-up time was calculated by the reverse Kaplan-Meier method.^20^ Statistical analyses used SPSS version 30.0 (IBM Corp., Armonk, NY), with *p*-values <0.05 considered significant differences.

## Results

### Characteristics of patients with CLAD

**Figure 1:**
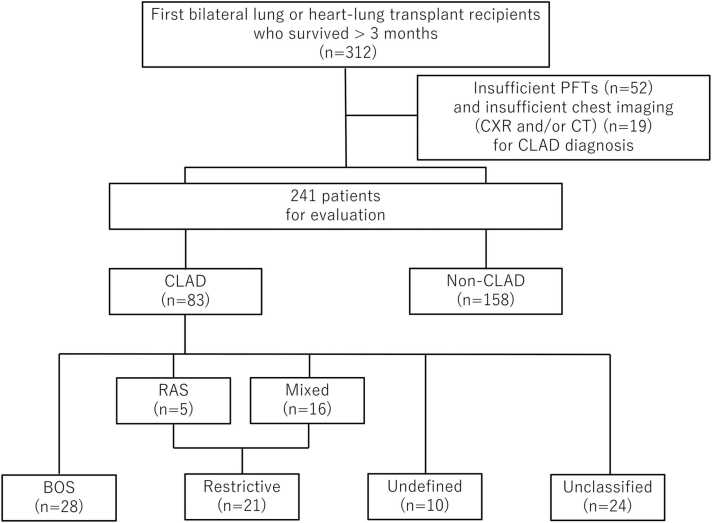
Flow diagram of the study cohort. BOS, bronchiolitis obliterans syndrome; CLAD, chronic lung allograft dysfunction; CT, computed tomography; CXR, chest x-ray; PFT, pulmonary function test; RAS, restrictive allograft syndrome.

Figure 1 represents the flow diagram of the study cohort. Of 312 first bilateral lung transplant recipients between January 2005 and December 2021, 52 were excluded due to insufficient PFTs data and 19 due to insufficient chest imaging. Among the 241 patients, 83 met the new ISHLT criteria and were included. Two patients in our cohort received heart-lung transplantation, but neither developed CLAD during the study period.

CLAD was divided into 4 categories: BOS, restrictive, undefined, and unclassified. Twenty-eight patients (33.7%) had BOS, 21 (25.3%) restrictive, 10 (12.0%) undefined, and 24 (29.0%) unclassified phenotype (Table 1). Baseline demographics and clinical characteristics showed no significant differences among groups.

**Table 1:** Patient Characteristics of CLAD Phenotypes

Characteristics	BOS	Restrictive	Undefined	Unclassified	*p*-value
Number of patients, *n* (%)	28 (33.7)	21 (25.3)	10 (12.0)	24 (29.0)	
Age at Tx, years, median (IQR)	55.5 (51.8-62.5)	54.0 (41.0-57.0)	52.5 (41.5-59.0)	59.0 (56.0-64.3)	0.078
Gender male, *n* (%)	17 (60.7)	10 (47.6)	4 (40.0)	14 (58.3)	0.61
Primary disease, *n* (%)					0.31
Pulmonary fibrosis	12 (42.9)	13 (61.9)	6 (60.0)	13 (54.2)	
COPD/emphysema	11 (39.3)	2 (9.5)	1 (10.0)	6 (25.0)	
Cystic fibrosis	3 (10.7)	4 (19.0)	3 (30.0)	2 (8.3)	
Other	2 (7.1)	2 (9.5)	0 (0.0)	3 (12.5)	
Time from Tx to CLAD months (IQR)	35.5 (15.6-54.1)	28.2 (21.7-48.4)	30.3 (20.7-71.5)	56.3 (32.0-68.7)	0.12

Abbreviations: BOS, bronchiolitis obliterans syndrome; CLAD, chronic lung allograft dysfunction; COPD, chronic obstructive pulmonary disease; IQR, interquartile range; Tx, transplant.

Supplemental Figure 1 illustrates the phenotypic distribution of CLAD based on combinations of obstructive and restrictive ventilatory defects with the presence or absence of RLOs. Out of 21 patients with restrictive phenotype, 5 had RAS and 16 had mixed phenotype. RLOs without restrictive ventilatory defects were observed in only 1 case, which was classified as undefined.

### Distribution pattern of RLOs in restrictive phenotype

**Figure 2:**
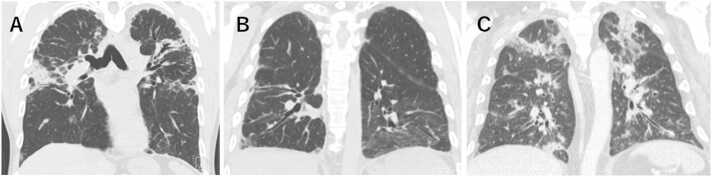
Distribution patterns of RAS-like opacities (RLOs) on CT in CLAD patients. (A) CT image demonstrating upper-predominant RLOs in a patient with a survival time of 5 years after CLAD diagnosis. (B) Lower-predominant RLOs with a survival time of 3 years after CLAD diagnosis. (C) Diffuse RLOs with a survival time of 5 months after CLAD diagnosis. CLAD, chronic lung allograft dysfunction; CT, computed tomography; RAS, restrictive allograft syndrome; RLOs, RAS-like opacities.

Among 21 patients of restrictive phenotype, the distribution of RLOs at the time of CLAD diagnosis was upper-predominant in 6 (28.6%), lower-predominant in 2 (9.5%), and diffuse distribution in 13 (61.9%) (Figure 2A-C). Detailed patient characteristics of these 3 groups are summarized in Supplemental Table 1.

### Survival analysis of CLAD phenotypes with distribution patterns of restrictive phenotype

Among the 83 patients diagnosed with CLAD, 42 either died (*n* = 40) or underwent retransplantation (*n* = 2), with a median time from CLAD diagnosis to death or retransplantation of 52.2 months (95% confidence interval [CI]: 37.4-67.1 months). The median follow-up duration was 40.0 months (95% CI: 32.5-47.4). Across CLAD phenotypes, patients with the restrictive phenotype exhibited the shortest median OS at 19.8 months (95% CI: 5.9-33.8), followed by the undefined (41.7 months; 95% CI: 12.2-71.3) and unclassified phenotypes (55.4 months; 95% CI: 21.7-89.0). Patients with BOS had the longest OS at 68.1 months (95% CI: 56.7-79.5) (log-rank test, *p* = 0.002; Supplemental Figure 2).

In a multivariable Cox proportional hazards model adjusted for age and sex, the restrictive phenotype was the only phenotype independently associated with shorter survival compared to BOS as the reference (hazard ratio [HR], 4.53; 95% CI: 1.96-10.49; *p* < 0.001) (Supplemental Table 2).

**Figure 3:**
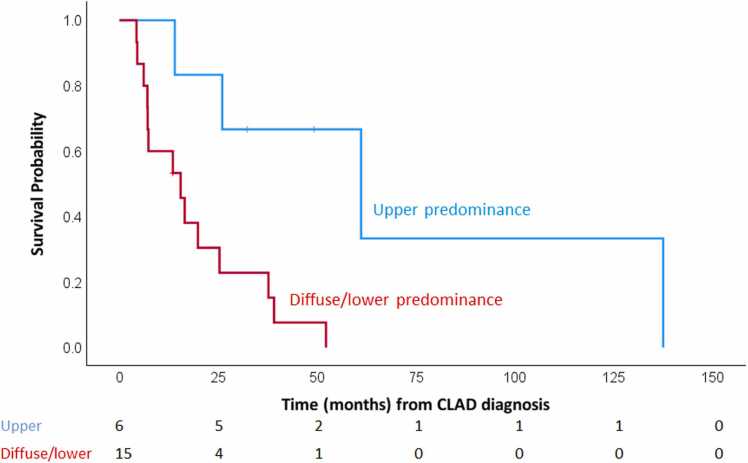
Kaplan-Meier survival curves comparing overall survival from the time of CLAD diagnosis between upper-predominant and diffuse/lower-predominant groups. Significantly shorter survival was observed in patients with diffuse/lower-predominant group compared with upper-predominant group (log-rank test, *p* = 0.008). CLAD, chronic lung allograft dysfunction.

Within the restrictive phenotype, survival significantly varied by RLO distribution pattern (log-rank test, *p* = 0.024; Supplemental Figure 3). Upper-predominant group showed a median OS of 61.1 months (95% CI: 8.5-113.7). Lower-predominant group (*n* = 2) showed a median OS of 15.5 months, and diffuse distribution group (*n* = 13) showed a median OS of 13.5 months (95% CI: 3.2-23.8). For exploratory analyses, subsequent analyses were performed comparing the upper-predominant group (*n* = 6) with the combined group of lower-predominant and diffuse distribution cases (*n* = 15). Patients in the upper-predominant group had significantly longer survival than those in the diffuse/lower-predominant group (median OS: 61.1 months; 95% CI: 8.5-113.7 vs 15.5 months; 95% CI: 4.7-26.2; *p* = 0.008; Figure 3). Detailed characteristics of these groups are summarized in Table 2.

**Table 2:** Patient Characteristics of Restrictive Phenotypes (Upper, Diffuse/Lower)

Characteristics	Upper	Diffuse/Lower	*p*-value
Number of patients, *n* (%)	6 (28.6)	15 (71.4)	
Age at Tx, years, median (IQR)	47.0 (39.3-54.8)	54.0 (43.5-59.0)	0.38
Gender male, *n* (%)	4 (66.7)	6 (40.0)	0.36
Primary disease, *n* (%)			0.47
Pulmonary fibrosis	4 (66.7)	9 (60.0)	
COPD/emphysema	0 (0.0)	2 (13.3)	
Cystic fibrosis	2 (33.3)	2 (13.3)	
Other	0 (0.0)	2 (13.3)	
Time from Tx to CLAD months (IQR)	43.7 (29.0-47.2)	26.9 (17.9-45.4)	0.21

Abbreviations: CLAD, chronic lung allograft dysfunction; COPD, chronic obstructive pulmonary disease; IQR, interquartile range; Tx, transplant.

**Figure 4:**
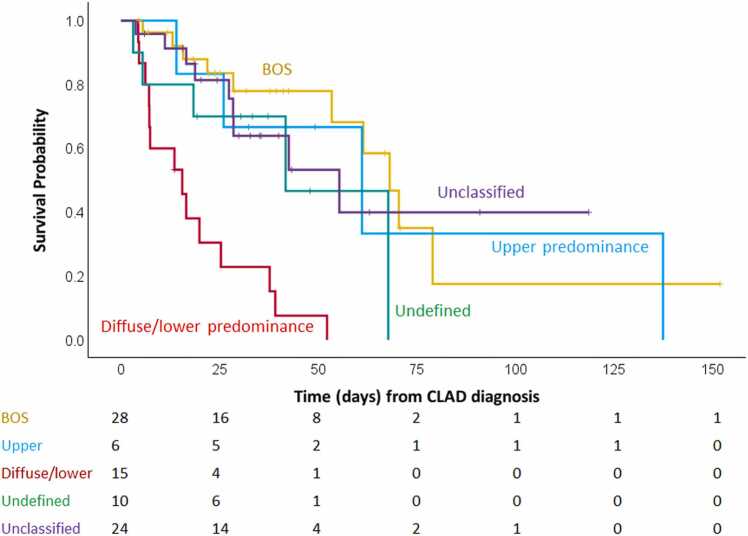
Survival analysis of CLAD phenotypes with restrictive phenotype subdivided into upper-predominant and diffuse/lower-predominant groups. Kaplan-Meier survival curves demonstrated statistically significant differences in overall survival from the time of CLAD diagnosis among CLAD phenotypes (log-rank test, *p* < 0.001). Patients in the diffuse/lower-predominant group showed shorter survival than patients with other phenotypes. BOS, bronchiolitis obliterans syndrome; CLAD, chronic lung allograft dysfunction.

When compared to other CLAD phenotypes, the diffuse/lower-predominant group was independently associated with significantly shorter survival in a multivariate Cox model adjusted for age and sex, using BOS as the reference (HR, 8.45; 95% CI: 3.40-21.04; *p* < 0.001). By contrast, the upper-predominant group showed no significant survival difference compared to nonrestrictive CLAD phenotypes (HR, 1.60; 95% CI: 0.45-5.62; *p* = 0.466) (Supplemental Table 3). Kaplan-Meier survival curves are presented in Figure 4.

### Semiquantitative analysis of CT findings at CLAD diagnosis in restrictive phenotype

Analysis of CT findings in restrictive phenotype cases showed significantly higher scores in the diffuse/lower-predominant group compared to the upper-predominant group for lower lung zone GGOs (2.13 ± 1.30 vs 0.83 ± 0.75, *p* = 0.030), lower lung zone reticular opacities (1.40 ± 0.63 vs 0.67 ± 0.52, *p* = 0.025), and lower lung zone RLOs (3.20 ± 1.15 vs 1.33 ± 1.03, *p* = 0.006). However, no significant differences were observed in the remaining radiological parameters, including total RLO scores between the groups (5.67 ± 2.26 vs 4.17 ± 2.14, *p* = 0.135) (Supplemental Table 4). Cox proportional hazards analysis demonstrated that the total RLO scores were not significantly associated with survival in either the upper-predominant (HR, 1.53; 95% CI: 0.83-2.82, *p* = 0.177) or diffuse/lower-predominant groups (HR, 0.87; 95% CI: 0.66-1.16, *p* = 0.337) (Supplemental Table 5).

### Radiological progression and pathological features according to initial CT patterns

Of 21 patients with restrictive phenotype, 20 patients had at least 1 follow-up CT scan after CLAD diagnosis (mean period: 21.3 ± 27.7 months).

**Figure 5:**
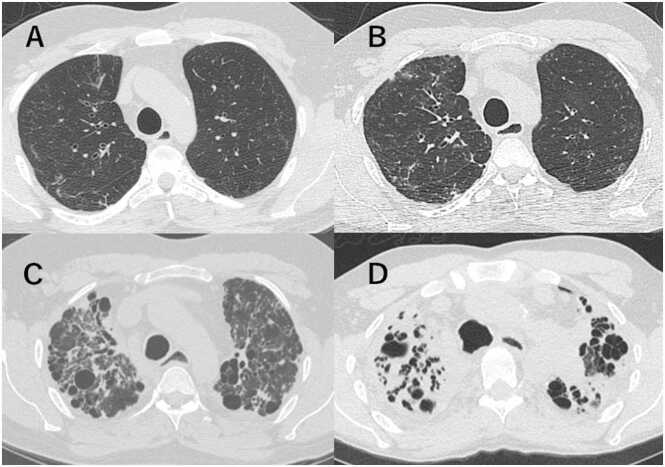
A 34-year-old male patient with an upper-predominant case underwent serial chest CT scans over a 10-year period. (A) Initial CT at CLAD diagnosis showed ground-glass opacities (GGOs), small linear and reticular opacities predominantly in the right upper lobe. (B) One year after diagnosis, a progressive increase in the right lung opacities was demonstrated, with new GGOs and small nodules appearing in the left upper lobe. (C) Five years after diagnosis, marked volume loss was revealed in both lungs, with the development of consolidation accompanied by bronchiectasis. (D) Ten years after diagnosis, dense consolidation was observed with bronchiectasis and cystic changes. Notable volume loss is evident in both upper lobes. The patient died 11.5 years after CLAD diagnosis. CLAD, chronic lung allograft dysfunction; CT, computed tomography.

Upper-predominant group initially included 6 patients, with 5 having CT follow-up. These 5 cases typically progressed from discrete nodular and reticular patterns with GGOs to increased reticular thickening. Four cases (4 of 5, 80%) developed consolidation with upper lung predominance, accompanied by cystic changes, bronchiectasis, and volume loss (Figure 5). An autopsy case demonstrated similar CT features to those of the 4 cases, with histopathology revealing patchy fibroelastosis most prominent in the bilateral upper lobes, consistent with findings of PPFE.

Lower-predominant cases (*n* = 2) showed initial consolidation or reticular opacities, progressing to diffuse distribution predominantly affecting the lower lungs with bronchiectasis and volume loss.

**Figure 6:**
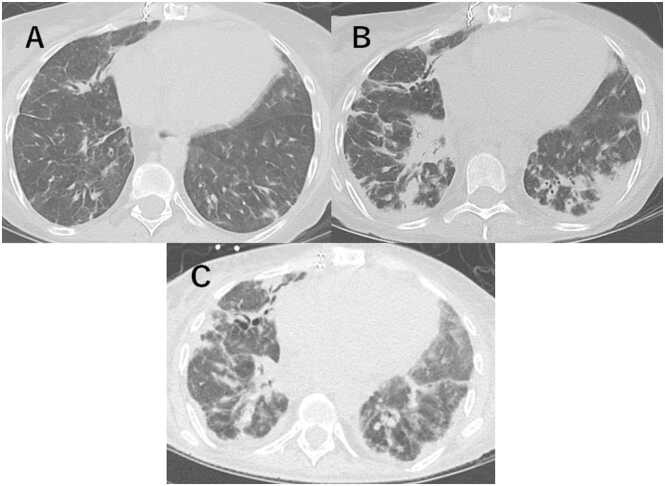
A 39-year-old male patient with a diffuse/lower-predominant case underwent serial chest CT scans over a 4-month period. (A) Initial CT at CLAD diagnosis showed diffuse ground-glass opacities (GGOs) with scattered areas of consolidation. (B) Three months after diagnosis, new consolidations appeared predominantly along the bronchovascular bundles. (C) Four months after diagnosis, extensive GGOs reappeared, indicating rapid progression, accompanied by bronchiectasis and decreased lung volume. The patient died 5 months after CLAD diagnosis. CLAD, chronic lung allograft dysfunction; CT, computed tomography.

Diffuse distribution cases (*n* = 13) frequently presented with diffuse GGOs and consolidation, which were more prevalent throughout the follow-up than in upper-predominant cases and showed 2 distinct initial patterns: 11 patients (84.6%) presented with diffuse GGOs and consolidation, while 2 patients (15.4%) predominantly exhibited reticular patterns. During follow-up, 2 patients (15.4%) developed upper lung predominant consolidation associated with volume loss. Seven patients (53.8%) showed persistent GGOs and consolidation, accompanied by bronchiectasis and volume loss (Figure 6). Of these 7 patients, 4 (30.8%) showed rapid progression of diffuse GGOs with consolidation after CLAD diagnosis. One patient from this rapid progression group (Figure 6) had TBB that revealed histopathological findings of acute and chronic inflammation, fibrin deposition, focal organization, and reactive pneumocyte atypia, consistent with AFOP. The other autopsy case revealed bilateral lung parenchyma containing acute and organizing pneumonia with DAD superimposed on pleural elastosis. Both patients died within 50 days of CLAD diagnosis. In our cohort, TBB was performed in 4 cases in total. While 1 case yielded a definitive histological diagnosis of AFOP as described above, the remaining 3 cases were inconclusive due to concurrent infection or inflammation.

## Discussion

This study aimed to predict survival outcomes based on the distribution patterns of RLOs at the time of CLAD diagnosis in patients with restrictive phenotypes, encompassing both RAS and mixed phenotypes as defined by the new ISHLT criteria. Our analysis combined qualitative assessment of RLO distribution patterns with semiquantitative evaluation to provide a thorough analysis of imaging features. This comprehensive approach integrated radiological findings with clinical outcomes and pathological correlates to evaluate their prognostic implications. To investigate the relationship between RLO distribution and outcomes, RLOs were categorized into 2 groups: upper-predominant and diffuse/lower-predominant. Our analysis suggests that patients in the diffuse/lower-predominant group showed a tendency toward worse prognoses compared to the upper-predominant group and exhibited the poorest outcomes across all CLAD phenotypes.

Among 241 patients with sufficient clinical data, 83 patients (34.4%) met the CLAD diagnostic criteria, with a phenotype distribution of 33.7% BOS and 25.3% restrictive phenotype, fell within the expected intercohort variation.^6^, ^21^ Importantly, the restrictive phenotype demonstrated a significantly worse prognosis compared to BOS, aligning with previous studies.^6^, ^8^ Both TLC/FVC decline and the presence of RLOs emerged as key diagnostic indicators for the restrictive phenotype, underscoring their role as poor prognostic markers^22^, ^23^; previous reports have shown that even undefined and unclassified cases with RLOs exhibited poor outcomes,^6^ though the limited sample size in our cohort precluded detailed analysis of these subgroups.

Prior studies have linked RLO distribution and progression patterns on CT with outcomes in restrictive phenotypes. For example, rapid progression of persistent CT abnormalities^11^ and diffuse inflammatory changes, such as consolidation and GGOs,^24^ were associated with shorter survival, whereas upper-predominant infiltrates were correlated with better prognoses.^10^ Our study builds on these insights by incorporating the updated ISHLT criteria and systematically comparing phenotypes, with the addition of semiquantitative assessment of RLO extent. In our cohort, we observed that patients with lower-predominant distribution (*n* = 2) showed median survival times comparable to those with diffuse distribution (*n* = 13). While previous studies have suggested potential similarities between these patterns,^10^ our limited sample size, particularly in the lower-predominant group, prevents definitive conclusions about whether these patterns represent distinct prognostic groups or share similar characteristics. This observation warrants further investigation in larger cohorts. Nevertheless, our findings, combined with previous studies investigating RLO distribution patterns in restrictive CLAD, contribute to the growing evidence that radiological distribution patterns at diagnosis may serve as important prognostic indicators. Using this comprehensive approach, we found that patients with diffuse/lower-predominant distribution were associated with poorer prognoses than all other CLAD phenotypes, although the extent of RLOs within each distribution pattern was not significantly associated with survival outcomes. This developing body of evidence suggests that careful evaluation of RLO distribution patterns at CLAD diagnosis could potentially help identify patients who may benefit from more aggressive therapeutic interventions, such as antifibrotic agents^25^, ^26^ and total lymphoid irradiation,^27^ or earlier consideration for retransplantation.^28^

Distinct CT findings and progression patterns emerged between upper-predominant and diffuse/lower-predominant groups. The upper-predominant group showed progressive consolidation and volume loss predominantly in upper lobes, radiologically suggestive of PPFE, with one autopsy case showing compatible histopathological findings.^16^, ^29^ In contrast, the diffuse/lower-predominant group tended to progress to diffuse involvement with 2 distinct patterns. First, in patients with diffuse distribution, extensive GGOs with consolidation developed in 4 cases requiring oxygen supplementation, consistent with acute exacerbations often seen in restrictive phenotypes.^9^, ^30^ Verleden et al described such presentations as “white-out” or late acute graft failure, characterized by diffuse pulmonary infiltrates, potentially corresponding to AFOP or DAD, which are histologically associated with poor outcomes.^17^ Pathological findings in our cohort supported this, with one autopsy case showing DAD and another biopsy-confirmed case of AFOP; both patients succumbed within 50 days. Second, diffuse/lower-predominant cases suggested progressive fibrosis across both lungs compared to upper-predominant cases, leading to reduced ventilatory capacity and survival.^31^ By contrast, the upper-predominant group’s favorable outcomes may partly stem from a lower incidence of DAD beyond 1 year post-CLAD diagnosis in PPFE-dominant cases.^16^

While previous studies have primarily relied on qualitative assessments of radiological patterns, we implemented both distribution-based classification and semiquantitative scoring of RLO extent. Given the limited sample size and to ensure robust statistical analysis, we focused on the total RLO scores rather than individual opacity patterns. Notably, the total RLO scores were not significantly associated with survival in either the upper-predominant or diffuse/lower-predominant groups. This lack of association between RLO extent and survival within each distribution pattern suggests that once the pattern is established (either upper-predominant or diffuse/lower-predominant), the extent of involvement does not further stratify prognosis. It raises the possibility that the type of pathological process, as indicated by the distribution pattern, appears to be more important than the quantitative extent of the disease. In the diffuse/lower-predominant group, the distribution pattern itself may indicate an aggressive pathological process, regardless of RLO extent, as these patients can develop acute exacerbations. In contrast, in the upper-predominant group, there was a trend toward shorter survival with higher total RLO scores, possibly reflecting a more uniform disease progression compared to the diffuse/lower-predominant group. However, further studies with larger cohorts are needed to fully characterize the relationship between RLO extent and outcomes within each distribution pattern.

This study has several limitations. First, it is based on a single cohort with limited cases, particularly in the lower-predominant group (*n* = 2). Although we observed similar survival outcomes between lower-predominant and diffuse distribution patterns, and previous studies have suggested potential similarities,^10^ our limited sample size in the lower-predominant group (*n* = 2) prevents us from drawing definitive conclusions about whether these patterns represent the same prognostic group. Additionally, while the pathological specimens provided valuable insights, their limited number prevents definitive conclusions about the underlying pathological findings. Second, both pure RAS and mixed phenotypes were included in the restrictive phenotype, as they demonstrate comparable prognoses,^32^ and both require RLOs for their diagnosis according to ISHLT criteria.^5^ Third, FVC was used as a surrogate for TLC,^5^, ^33^ potentially causing misclassification since BOS can also show FVC decline.^8^ To address this, phenotypes were classified using a combination of functional and radiological parameters. Fourth, single-lung transplant recipients were excluded due to the complexities of interpreting 2-compartment pulmonary function changes.^7^ Finally, external validation across multicenter cohorts is needed to confirm these findings.

In conclusion, diffuse/lower-predominant distribution of RLOs appeared to be associated with a significantly worse prognosis compared to upper-predominant pattern in restrictive phenotype CLAD. Moreover, the diffuse/lower-predominant group suggested poorer outcomes compared to other CLAD phenotypes. Notably, the extent of RLOs within these distribution groups did not correlate with survival. While our limited sample size precludes definitive conclusions, our findings add to the growing evidence suggesting CT distribution patterns may help predict outcomes in CLAD patients.

## CRediT authorship contribution statement

Conceptualization: T.F., H.H.; Methodology: T.F., H.H.; Formal analysis: T.F., Y.L.; Investigation: T.F., Y.N., S.T., M.N., H.H; Resources Data: T.F., S.T., M.N.; Data curation: T.F., S.T., M.N.; Writing - original draft: T.F.; Writing - review and editing: T.F., Y.N., S.T., Y.K., S.M.G., T.J., Y.L., D.C.C., H.O., L.S., M.N., H.H.; Visualization: T.F., Y.K., H.H.; Supervision: M.N., H.H.

## Disclosure statement

Y.L. has received funding from an 10.13039/100000002NIH (10.13039/100000054NCI) grant (R01-CA249096) outside of the submitted work. D.C.C. has received funding from an NIH (NCI) grant (U01-CA209414) outside of the submitted work. L.S. has received funding from 10.13039/100004328Genentech and 10.13039/100008021Bristol Myers Squibb, and consulting fees from Genentech and Lilly, all outside of the submitted work. M.N. has received a research grant to the institution from 10.13039/100015650Canon Medical Systems and Konica Minolta Inc., and consulting fees from AstraZeneca, all outside of the submitted work. H.H. has received a research grant from Canon Medical Systems Inc. and Konica Minolta Inc., consulting fees from Canon Medical Systems Inc. and Boehringer Ingelheim Inc., and a provisional US patent application (63/610,842), all outside of the submitted work. The remaining authors have no relevant disclosures.

The authors declare no acknowledgments applicable to this work.

## Footnotes

Appendix A Supplementary data associated with this article can be found in the online version at doi:10.1016/j.jhlto.2025.100232.
