# Supplementary material for: Radiological distribution patterns in restrictive chronic lung allograft dysfunction: Impact on survival across all phenotypes
Source: JHLT Open. 2025 Feb 18;8:100232. doi: 10.1016/j.jhlto.2025.100232 (PMC11935435; doi:10.1016/j.jhlto.2025.100232)
Supplement: Supplementary file 1 — Supplementary material [file mmc1.docx]

**Supplemental Figure legend**

**Supplemental Figure 1**

Combinations of obstructive and restrictive ventilatory defects with the presence or absence of RLOs in CLAD phenotypes. Pulmonary function is assessed according to FEV_1_/FVC (%) to indicate obstructive defects (< 70%) and FVC (%) to indicate restrictive defects (≤ 80%). The number of cases (N) is shown in each quadrant. (A) Cases with RLOs are classified into restrictive (RAS, mixed), undefined, and unclassified phenotypes. (B) Cases without RLOs are categorized into BOS, undefined, and unclassified phenotypes. BOS, bronchiolitis obliterans syndrome; CLAD, chronic lung allograft dysfunction; FEV_1_, forced expiratory volume in one second; FVC: forced vital capacity; RAS, restrictive allograft syndrome; RLOs, RAS-like opacities.

**Supplemental Figure 2**

Survival analysis based on CLAD phenotypes. Kaplan-Meier survival curves demonstrated statistically significant differences in overall survival from the time of CLAD diagnosis among CLAD phenotypes (log-rank test, *P* = 0.002). Patients with restrictive phenotype had shorter survival than patients with other phenotypes. BOS, bronchiolitis obliterans syndrome; CLAD, chronic lung allograft dysfunction.

**Supplemental Figure 3**

Kaplan-Meier survival curves comparing overall survival from the time of CLAD diagnosis among three restrictive phenotype groups. The difference among groups was statistically significant (log-rank test, *P* = 0.024), and the upper-predominant group showed longer survival. CLAD, chronic lung allograft dysfunction.
