# Supplementary material for: Radiological distribution patterns in restrictive chronic lung allograft dysfunction: Impact on survival across all phenotypes
Source: JHLT Open. 2025 Feb 18;8:100232. doi: 10.1016/j.jhlto.2025.100232 (PMC11935435; doi:10.1016/j.jhlto.2025.100232)
Supplement: Supplementary file 2 — Supplementary material [file mmc2.docx]

**Supplemental Table 1**

Patient characteristics of restrictive phenotypes (Upper, Lower, Diffuse)

| Characteristics | Upper | Lower | Diffuse |
| --- | --- | --- | --- |
| Number of patients, n (%) | 6 (28.6) | 2 (9.5) | 13 (61.9) |
| Age at Tx, years, median (IQR) | 47.0 (39.3-54.8) | 51.0 (44.0-58.0) | 57.0 (43.0-60.0) |
| Gender male, n (%) | 4 (66.7) | 2 (100.0) | 4 (30.8) |
| Primary disease, n (%) |  |  |  |
| Pulmonary fibrosis | 4 (66.7) | 1 (50.0) | 8 (61.5) |
| COPD/emphysema | 0 (0.0) | 1 (50.0) | 1 (7.7) |
| Cystic fibrosis | 2 (33.3) | 0 (0.0) | 2 (15.4) |
| Other | 0 (0.0) | 0 (0.0) | 2 (15.4) |
| Time from Tx to CLAD months (IQR) | 43.7 (29.0-47.2) | 45.2 (29.7-60.6) | 22.0 (16.0-37.3) |

Abbreviations: CLAD, chronic lung allograft dysfunction; COPD, chronic obstructive pulmonary disease; IQR, interquartile range; Tx, transplant.

**Supplemental Table 2**

Multivariable Cox proportional hazards models evaluating CLAD phenotypes with time from CLAD diagnosis to death/retransplant.

| Phenotype | Hazard ratio | 95% CI | *P* -value |
| --- | --- | --- | --- |
| Restrictive | 4.53 | 1.96-10.49 | < 0.001 |
| Undefined | 2.15 | 0.70-6.59 | 0.181 |
| Unclassified | 1.34 | 0.54-3.33 | 0.534 |

Abbreviations: CI, confidence intervals; CLAD, chronic lung allograft dysfunction.

Bronchiolitis obliterans syndrome was used as the reference category. Adjusted for age at transplant and sex.

**Supplemental Table 3**

Multivariable Cox proportional hazards models evaluating CLAD phenotypes with restrictive phenotype subdivided into upper-predominant and diffuse/lower-predominant groups with time from CLAD diagnosis to death/retransplant.

| Phenotype | Hazard ratio | 95% CI | *P* -value |
| --- | --- | --- | --- |
| Upper | 1.60 | 0.45-5.62 | 0.466 |
| Diffuse/Lower | 8.45 | 3.40-21.04 | < 0.001 |
| Undefined | 2.14 | 0.69-6.63 | 0.188 |
| Unclassified | 1.39 | 0.55-3.47 | 0.486 |

Abbreviations: CI, confidence intervals; CLAD, chronic lung allograft dysfunction.

Bronchiolitis obliterans syndrome was used as the reference category. Adjusted for age at transplant and sex.

**Supplemental Table 4**

Semi-quantitative analysis of CT findings between upper-predominant and diffuse/lower-predominant groups at CLAD diagnosis

| Characteristics | Upper predominance | Diffuse/Lower predominance | *P* -value |
| --- | --- | --- | --- |
| GGOs (Upper) | 1.67 ± 0.82 | 1.67 ± 1.11 | 0.967 |
| GGOs (Lower) | 0.83 ± 0.75 | 2.13 ± 1.30 | 0.030 |
| Consolidation (Upper) | 0.67 ± 1.03 | 0.53 ± 0.74 | 0.928 |
| Consolidation (Lower) | 0.17 ± 0.41 | 0.67 ± 0.62 | 0.078 |
| Reticular opacities (Upper) | 1.50 ± 0.84 | 1.13 ± 0.64 | 0.418 |
| Reticular opacities (Lower) | 0.67 ± 0.52 | 1.40 ± 0.63 | 0.025 |
| Upper RLOs | 2.83 ± 1.17 | 2.47 ± 1.19 | 0.746 |
| Lower RLOs | 1.33 ± 1.03 | 3.20 ± 1.15 | 0.006 |
| Total RLOs | 4.17 ± 2.14 | 5.67 ± 2.26 | 0.135 |

Abbreviations: CLAD, chronic lung allograft dysfunction; GGOs, ground-glass opacities; RLOs, RAS (restrictive allograft syndrome)-like opacities.

Data are means ± SDs. Mann-Whitney U tests were used to compare semi-quantitative CT scores between upper-predominant and diffuse/lower-predominant groups at CLAD diagnosis.
Each radiological finding was scored on a 6-point scale (0-5) for upper and lower lung zones separately: 0, no involvement; 1, less than 5%; 2, 5%-25%; 3, 26%-49%; 4, 50%-75%; and 5, greater than 75% involvement. Upper and Lower RLOs (GGOs, consolidation, or reticular opacities) were scored separately by evaluating the total area affected by any type of RLO in each lung zone. Total RLOs represent the sum of Upper and Lower RLO scores.

**Supplemental Table 5**

Cox proportional hazards models evaluating total RLO score in relation to time from CLAD diagnosis to death/retransplant in upper-predominant and diffuse/lower-predominant groups.

| Phenotype | Hazard ratio | 95% CI | *P* -value |
| --- | --- | --- | --- |
| Upper predominant | 1.53 | 0.83-2.82 | 0.177 |
| Diffuse/Lower predominant | 0.87 | 0.66-1.16 | 0.337 |

Abbreviations: CI, confidence interval; CLAD, chronic lung allograft dysfunction; RLO, RAS (restrictive allograft syndrome)-like opacity.

The hazard ratio (HR) represents the change in hazard per 1-point increase in the total RLO score.
