# Supplementary figures and images for: Radiological distribution patterns in restrictive chronic lung allograft dysfunction: Impact on survival across all phenotypes
Source: JHLT Open. 2025 Feb 18;8:100232. doi: 10.1016/j.jhlto.2025.100232 (PMC11935435; doi:10.1016/j.jhlto.2025.100232)

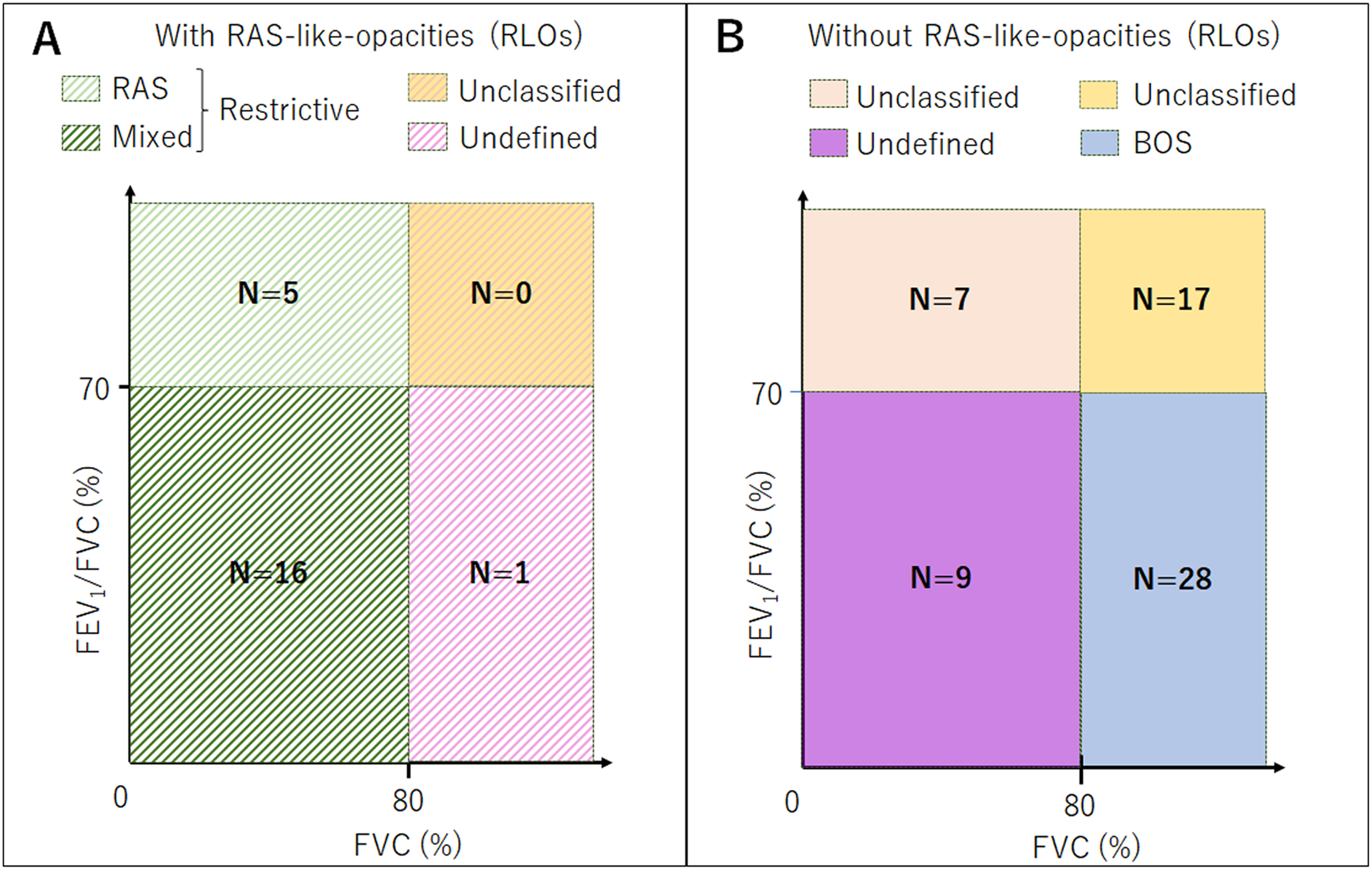

Supplement: Supplementary file 3 — Supplementary material [file mmc3.jpg]

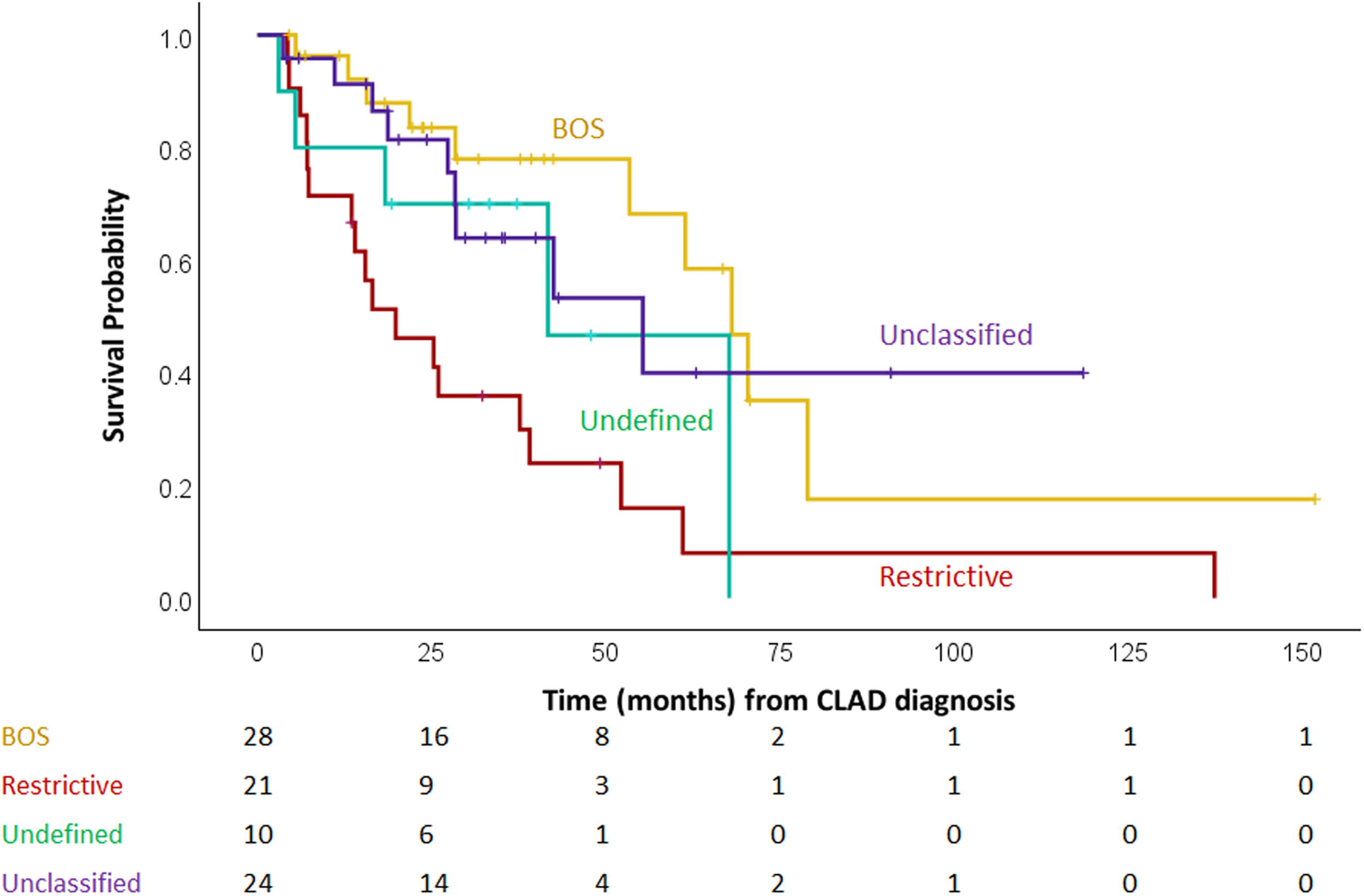

Supplement: Supplementary file 4 — Supplementary material [file mmc4.jpg]

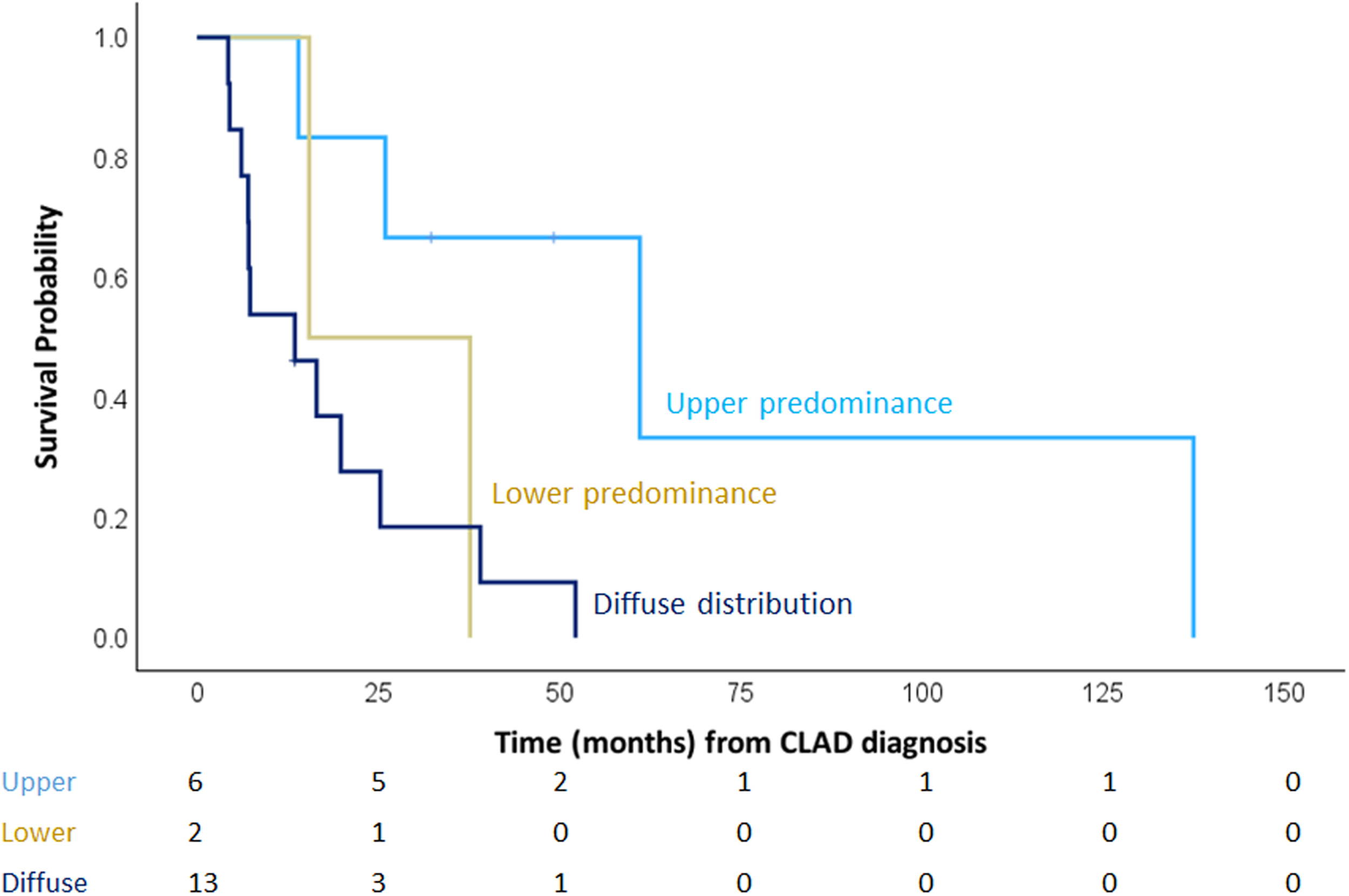

Supplement: Supplementary file 5 — Supplementary material [file mmc5.jpg]
